# 515. Comparing Different Delivery Methods of Fecal Microbiota Transplantation

**DOI:** 10.1093/ofid/ofac492.571

**Published:** 2022-12-15

**Authors:** Eun Hwa Lee, Ki Hyun Lee, Jinnam Kim, Se Ju Lee, Jung Ho Kim, Jin Young Ahn, Su Jin Jeong, Nam Su Ku, Joon-sup Yeom, Jun Yong Choi

**Affiliations:** Yonsei University College of Medicine, Seoul, Seoul-t'ukpyolsi, Republic of Korea; Yonsei University College of Medicine, Seoul, Seoul-t'ukpyolsi, Republic of Korea; Yonsei University College of Medicine, Seoul, Seoul-t'ukpyolsi, Republic of Korea; Inha University College of Medicine, Seoul, Seoul-t'ukpyolsi, Republic of Korea; Yonsei University College of Medicine, Seoul, Seoul-t'ukpyolsi, Republic of Korea; Yonsei University College of Medicine, Seoul, Seoul-t'ukpyolsi, Republic of Korea; Yonsei University College of Medicine, Seoul, Seoul-t'ukpyolsi, Republic of Korea; Division of Infectious Diseases, Department of Internal Medicine, Yonsei University College of Medicine, Seoul, Seoul-t'ukpyolsi, Republic of Korea; Division of Infectious Diseases, Department of Internal Medicine, Yonsei University College of Medicine, Seoul, Seoul-t'ukpyolsi, Republic of Korea; Yonsei University College of Medicine, Seoul, Seoul-t'ukpyolsi, Republic of Korea

## Abstract

**Background:**

The increasing prevalence of multi-drug resistant organism (MDRO) carriage imposes significant medical challenges by increasing healthcare costs. Since MDRO carriage impacts treatment outcome and prognosis, many decolonization strategies were proposed but were not as effective as expected. Recently, fecal microbiota transplantation (FMT) has been discussed as a novel and effective method of MDRO decolonization. In this study, we compared the effectiveness of different FMT delivery methods to find suitable methods and optimize the success rate of decolonization for the patients with MDRO carriage.

**Figure:**
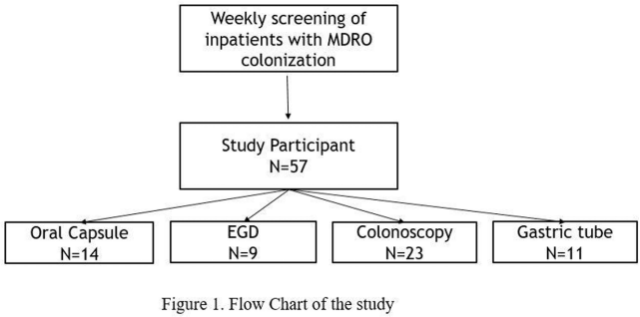

**Methods:**

In this prospective cohort study, we enrolled patients with MDRO carriage from 2018 to 2021. Patients received FMT via one of the four following methods; oral capsule, esophagogastroduodenoscopy, colonoscopy, or gastric tube. FMT delivery method was chosen according to the medical condition or eligibility for oral diet. The participants were followed up for one year. Decolonization and recolonization with MDRO were determined by the follow-up stool cultures. Microbiome analysis was done to assess the successful restoration of the gut microbiome.

**Results:**

A total of 57 patients received FMT for MDRO decolonization. The number of patients who received FMT via oral capsule, EGD, colonoscopy, and gastric tubes was 14, 9, 23, and 11. The overall decolonization rate was 69.2%, and the recolonization rate was 19.3%. The colonoscopy group required the shortest time for decolonization, while the EGD group was the longest (24.9 vs. 190.4 days, p-value=0.022). The decolonization rate was the highest in the EGD group (85.7%) and the lowest in the gastric tube group (50.5%). The oral capsule group had a comparable decolonization rate to the EGD group (84.6% vs. 85.7%, p=0.730). The clinical factor associated with decolonization was antibiotic usage after FMT (Odds ratio= 6.810, p-value=0.008). Microbiome analysis data showed improvement in alpha index in the Oral capsule and the EGD group. All four groups showed a reduced proportion of MDRO species after FMT.

**Figure:**
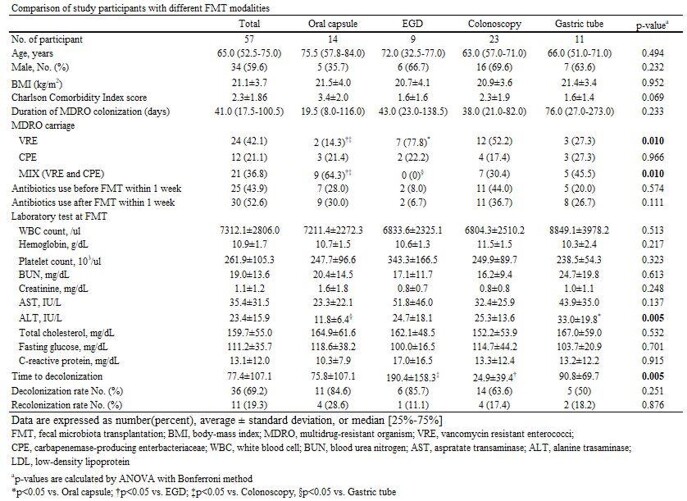

**Figure:**
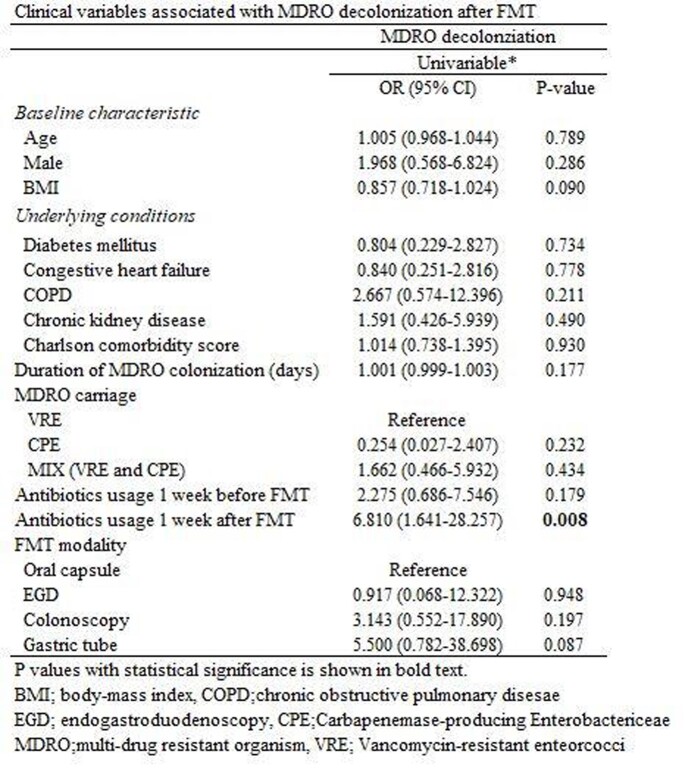

Comparison of ACE index of Taxonomic data with different FMT modalities

**Figure:**
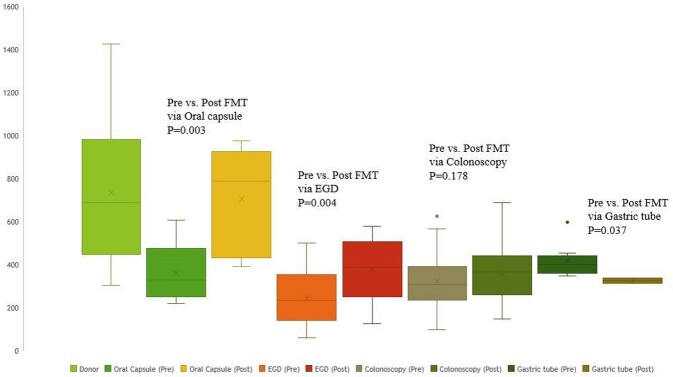

**Conclusion:**

The oral capsule is an effective FMT method for patients who are tolerable to an oral diet compared to the other conventional methods. Discontinuation of antibiotics after FMT is critical in decolonization.

**Disclosures:**

**All Authors**: No reported disclosures.

